# Parents’ Assessment of Their Children’s Use of Technology

**DOI:** 10.1192/j.eurpsy.2024.1185

**Published:** 2024-08-27

**Authors:** M. Tepetaş, A. Ünsal, G. S. Tepetaş Cengiz

**Affiliations:** ^1^Medical Faculty, Eskişehir Osmangazi University, Eskişehir; ^2^Mehmet Tanrıkulu Health Services Vocational School, Bolu Abant İzzet Baysal University, Bolu, Türkiye

## Abstract

**Introduction:**

The use of technology in many areas of daily life is widespread among both children and adults. Excessive and inappropriate use of technological aids causes significant problems in physical, psychomotor, psychological and social stages of development, especially in childhood. One of the reasons for some problems that arise in adulthood, such as communication problems, anxiety disorders, obesity, musculoskeletal disorders and tendency to violence is the excessive and inappropriate use of technology in childhood. For this reason, it is of great importance to complete the developmental stages in childhood in a healthy way.

**Objectives:**

The aim of this study was to qualitatively assess the technology use habits of 48-72 month old preschool children and their parents.

**Methods:**

The study is a qualitative research conducted among the parents of children in Eskişehir and Bolu between March and June 2023. A semi-structured form was used for the personal interviews with the 25 parents who constituted the study group. The interviews were recorded. The audio recordings were then transcribed and a thematic content analysis was conducted. The main themes of the interviews concerned the habits of parents’ and children’s in the use of technology at home, the content used on technological devices and how it is controlled, and the arrangements for technology use at home.

**Results:**

In the interviews, parents reported that when they needed to use technological devices, they most often chose a time and place when the children were not present or asleep. When children spent more time at home, this was the most common reason for increased technology use, while the most common reason for decreased use was that children spent more time outside the home. Most parents limited the amount of time their children’s daily technology using time. It was found that children generally complied with these restrictions, and when they did not, they often expressed themselves with reactions such as sulking/angry/crying.

**Conclusions:**

This study emphasizes that the most important factor determining children’s attitudes towards technology use is their parents’ attitudes towards technology use In order for children to develop a positive attitude towards technology use, it can be beneficial for parents to regulate and control their children’s technology use as well as their own.

**Disclosure of Interest:**

None Declared

